# The effects of emotional intelligence training programs on educators: a systematic review

**DOI:** 10.12688/f1000research.166190.1

**Published:** 2025-08-29

**Authors:** Myriam Liz Aponte, Ana Dolores Vargas Sánchez, Marco Arnao Vasquez

**Affiliations:** 1 Universidad de La Sabana, Chia, Cundinamarca, Colombia; 2 Universidad Catolica Santo Toribio de Mogrovejo, Chiclayo, Lambayeque, Peru

**Keywords:** Emotional Intelligence, Social Emotional Learning, teacher training, education

## Abstract

This article examines the evolution of emotional intelligence (EI) in teacher training using a systematic assessment of 67 publications published from 2013 to 2024 in the Scopus and Web of Science databases. The primary purpose is to identify ways and programs designed to enhance the emotional competences of educators. The evaluation, adhering to PRISMA principles, includes a bibliometric analysis that facilitates the assessment of scientific trends, geographical distribution of output, and strategic subjects within the discipline. The findings indicate that the most efficacious programs integrate Social-Emotional Learning (SEL), mindfulness techniques, emotional journaling, and virtual simulations, fostering competencies such as emotional self-regulation, empathy, and stress management. While short-term and adaptable programs demonstrate immediate advantages, additional research is required about their long-term viability. The virtualization of training in emotional intelligence and the utilization of reflecting technologies are prominent rising themes. The United States and Spain comprise the majority of the research, but Latin America exhibits minimal involvement, underscoring the necessity for contextualized approaches. Similarly, scientific collaboration networks and approved instruments for the assessment of emotional intelligence, such as EQ-i, MSCEIT, and TMMS, are recognized. The study underscores the significance of accompanying tactics and learning communities to guarantee the continuity and efficacy of the programs. In conclusion, a comprehensive, integrated, technologically facilitated, and context-aware strategy is recommended to improve educational quality. This evaluation provides an organized perspective on effective models and existing deficiencies, directing future research and program development.

## 1. Introduction

Throughout the 21st century, particularly during the health crisis induced by the COVID-19 pandemic, new roles for teachers have emerged in response to the evolving challenges faced by students, alongside advancements in knowledge, notably in neuroscience and emotional intelligence. ^1– 4^ This scenario has heightened the challenges and expectations placed on teachers, who are now required to address not only academic, technological, and pedagogical aspects but also the development of socioemotional skills, which are essential for effective classroom management and the holistic education of students. ^5– 8^

Educational processes are presently encountering new risks and challenges within the context of the fourth industrial revolution, marked by the integration of advanced technologies including artificial intelligence, the internet, robotics, augmented reality, and machine learning. ^9, 10^ The integration of these technologies has significantly altered the educational landscape, necessitating new competencies for modern educators. This evolution presents challenges from two angles: the advancement of Technologies for Learning and Knowledge (TAC) and the potential risks impacting behavior and emotional well-being. ^11^

Emerging technologies present both opportunities and challenges for learning and teaching. Teachers must cultivate emotional skills and competencies to adeptly manage an environment characterized by the pervasive influence of technology, particularly in contexts that impact well-being, such as cyberbullying, grooming, and phubbing. ^12, 13^ Emotional Intelligence (EI) is pertinent in this context, as its cultivation during university education or teacher training is crucial for the individual’s well-being and professional performance.

This article aims to analyze the evolution of research in global literature regarding strategies and programs for developing Emotional Intelligence ^14^ in teacher education through a systematic literature review covering the period from 2013 to 2024. This study aims to address the following research questions:

• What techniques and programs have been used for teacher training in emotional intelligence from 2013 to 2024?
• What are the defining features of an EI program for educator training?

### 1.1 Concepts of emotional intelligence

Reference [ 15] posits that the notion of Emotional Intelligence (EI) emerged from Thorndike’s concept of social intelligence in 1920 and Gardner’s multiple intelligences theory in 1983. Salovey and Mayer officially introduced the phrase in 1990, defining it as the ability to perceive, utilize, comprehend, and regulate emotions. In 1995, Goleman expanded the definition to include aspects like motivation and social skills. ^16^

The neurological basis of emotional intelligence, as proposed by LeDoux (1996), ^17^ involves the interaction between the amygdala and the neocortex, which supports emotional regulation in cognitive and social processes. ^18^ This neuroscientific perspective supports the idea that emotional intelligence (EI) is a skill that can be developed and improved. ^19^

This field has evolved through various models, including the ability model proposed by Mayer and Salovey ^20^ the trait model by Petrides and Furnham, ^21^ and Bar-On’s ^22^ emotional quotient. However, discussions regarding the validity and measurement of these models persist.

Fernández-Berrocal and Extremera ^23^ propose a comprehensive approach that emphasizes the dual role of emotional intelligence in managing emotions and optimizing cognitive processes and personal development. ^24^ Subsequently, they contributed to emotional intelligence research within the Hispanic context by adapting and validating measurement instruments and examining their application in educational environments.

Mayer et al. ^25^ refined their four-branch model, comprising (1) emotional perception, (2) emotional facilitation of thinking, (3) emotional understanding, and (4) emotional regulation. Bar-On ^22^ highlights the relationship between emotional and social competencies, whereas Petrides ^21^ characterize emotional intelligence as a personality trait. ^26^

Emotional intelligence has become increasingly recognized as a crucial competency for educators within the educational framework. Vesely-Maillefer and Saklofske ^27^ indicate that emotional intelligence (EI) in educators encompasses the regulation of personal emotions and the establishment of emotionally intelligent classroom environments, facilitating the holistic development of both educators and students. ^28^ In the digital era, amid the growing demands of teacher training, this skill is essential for enhancing teachers’ personal well-being and professional satisfaction, as well as for reducing work-related stress and promoting students’ socioemotional learning. ^29, 30^

Social and Emotional Learning (SEL) offers a structured educational framework aimed at enhancing emotional and social competencies in individuals of all ages. CASEL, established by Daniel Goleman and Eileen Rockefeller Growald, has delineated five fundamental SEL competencies: self-awareness, self-management, social awareness, relationship skills, and responsible decision-making. ^31^ The RULER approach developed by the Yale Center for Emotional Intelligence emphasizes the significance of recognizing, understanding, labeling, expressing, and regulating emotions. This framework advocates for the integration of social and emotional learning (SEL) in educational settings to enhance the well-being of all participants. ^32^

Mindfulness initiatives in education, as advocated by Jennings, Roeser, and Lantieri, ^33^ alongside the principles of positive psychology proposed by Duckworth et al., ^34^ have incorporated mindfulness practices into social-emotional learning (SEL), emphasizing their effects on emotional regulation and teacher well-being. This viewpoint is substantiated by affective neuroscience, ^35^ which provides a scientific foundation for comprehending the neural networks engaged in these practices. These elements are essential for 21st-century education, as the cultivation of socioemotional skills and mindfulness corresponds with the requirements of digital learning and artificial intelligence in the classroom, promoting an emotionally intelligent and adaptive educational environment.

The integration of emotional intelligence, social-emotional learning, and mindfulness in teacher training underscores a comprehensive approach that addresses current educational demands, enhancing both teacher well-being and the quality of the teaching environment in response to modern challenges.

## 2. Methods

This systematic review article was developed by adhering to the PRISMA (Preferred Reporting Items for Systematic Reviews and Meta-Analyses) guidelines, which served as the primary framework for the information search and analysis process. The subsequent steps were implemented: 1) Definition of eligibility criteria for study review; 2) Selection of online databases; 3) Definition of a search string for selected databases, selection of investigations, and presentation of results.

The databases chosen for this search were Scopus and Web of Science, as they ensure quality, depth, and breadth in the analysis of this review. Both databases are recognized as leaders in the academic domain, attributed to their comprehensive thematic coverage, rigorous journal selection, and continuous updates, ensuring access to the latest literature. Furthermore, they offer various bibliometric metrics and indicators that facilitate the assessment of scientific output. ^36, 37^

Search keywords were established based on the questions posed for application to the databases. The terms utilized included: emotional intelligence, teacher training, teacher professional development, SEL, preservice teacher education, and inservice teacher education (see Table 1).

**Table 1.** Framework of the search string for systematic mapping.

DataBase	String search
**Scopus**	(Emotional Intelligence OR Emotion Intelligence OR EI OR Social-Emotional Learning OR SEL OR Emotional Competence OR Emotional Quotient) AND (Inservice Teacher Education OR Preservice Teacher Education OR Teacher Education OR Educator Training OR Educator Education OR Teacher Professional Development OR Teacher Preparation)
**Web of science**	(Emotional Intelligence OR Emotion Intelligence OR EI OR Social-Emotional Learning OR SEL OR Emotional Competence OR Emotional Quotient) AND (Inservice Teacher Education OR Preservice Teacher Education OR Teacher Education OR Educator Training OR Educator Education OR Teacher Professional Development OR Teacher Preparation)

**Figure 1.**
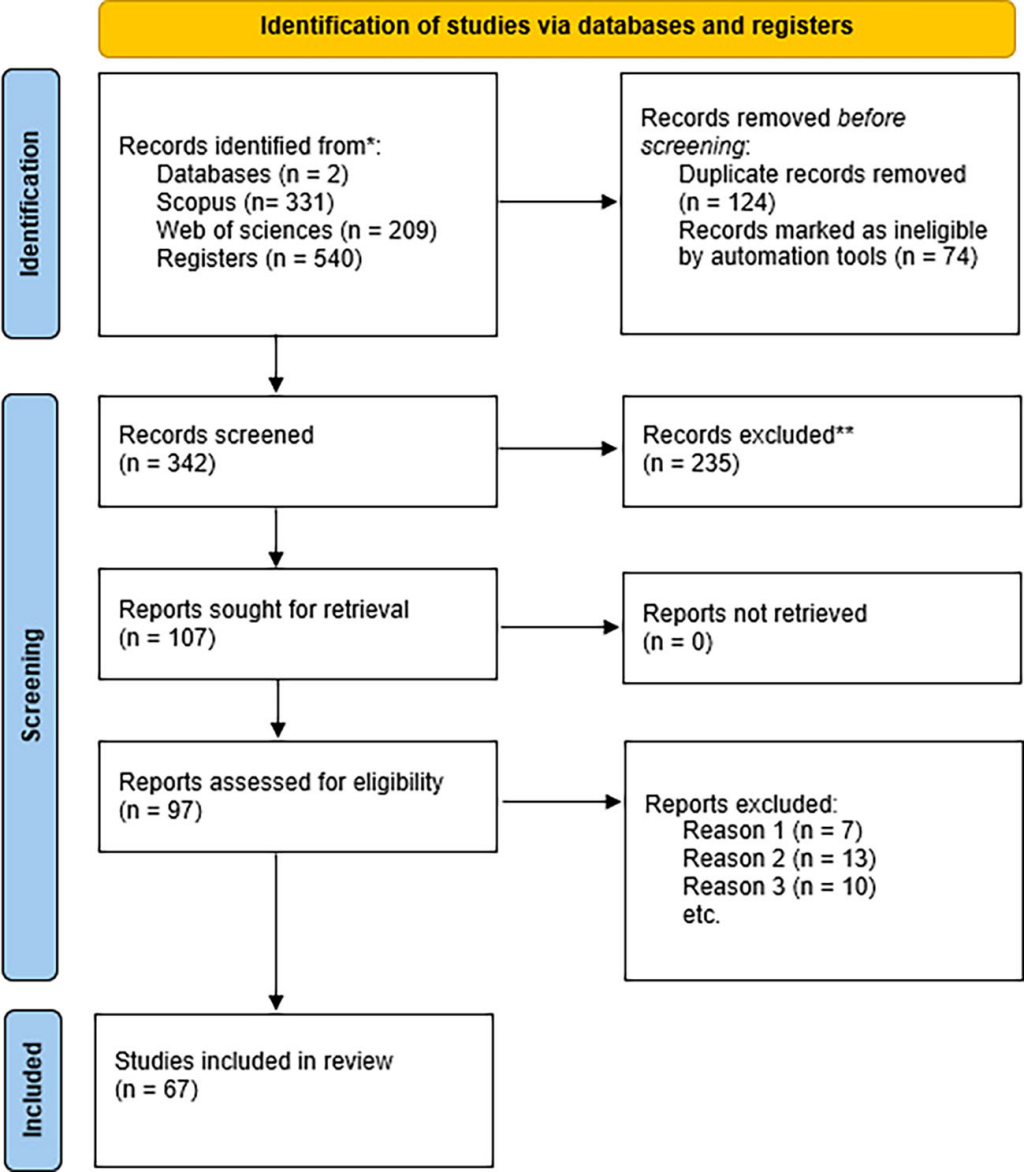
PRISMA flow diagram detailing steps in the phase of systematic review.

The literature search and study selection were conducted through several distinct steps. A total of 331 articles were identified in Scopus and 209 in Web of Science through the search strings employed. One hundred fifteen duplicate records were removed, and seventy-one were eliminated through the application of database filters. A total of 342 studies were evaluated through a review of their titles and abstracts according to the eligibility criteria, resulting in the exclusion of 235 records (see Figure 1).

### 2.1 Extraction and analysis of data

To ensure article relevance and quality, the following inclusion criteria were used: (1) Studies from 2013-2024. (2) Quantitative and/or qualitative research on emotional intelligence (EI) education or training programs and practices for pre-service or in-service teachers ensures a complete understanding of their influence. (3) Articles with actual data on program or strategy efficacy are included. Four studies involve pre-service or in-service instructors in educational intervention. The data from the literature review process, strategies and programs for developing Emotional Intelligence, the PRISMA checkboard and the process flow diagram have been published by Ref. [ 38] and are located in the availability section.

The article exclusion process occurred in multiple stages. Out of 235 articles, 107 were excluded from review to identify potentially relevant studies based on the established criteria. The selected articles (n = 97) underwent a thorough evaluation involving a comprehensive reading and analysis of each document to ascertain their relevance and quality. Theoretical studies lacking empirical data, intervention, or a focus on the teaching population in training or practice were excluded from consideration.

The analysis of the final articles (n = 67) (see Table 2) involved a bibliometric review utilizing bibliometrix, a scientific mapping and analysis tool that employs quantitative and descriptive techniques to assess academic production. This approach yields indicators related to regional production, prominent authors, strategic topics, key concepts, and significant collaboration nodes. ^39^ This analysis offers a comprehensive overview of the existing research landscape in this field.

**Table 2.** Annual article count.

*Year*	*Article*	*Year*	* Article*
2013	1	2019	9
2014	2	2020	9
2015	5	2021	6
2016	2	2022	11
2017	3	2023	8
2018	7	2024	4

To enhance the validity and reliability of the findings, qualitative and quantitative data from each article were organized in a matrix, facilitating the identification of strategies and programs developed for promoting emotional intelligence in teacher training. This record enabled the identification of their defining elements. Ethical approval is not required for this article.

## 3. Results

This section outlines the results regarding the growth trajectory, geographic distribution, and intellectual structure of the knowledge base on emotional intelligence in teacher education.

### 3.1 Global overview of scientific production in teacher education within early intervention

**Figure 2.**
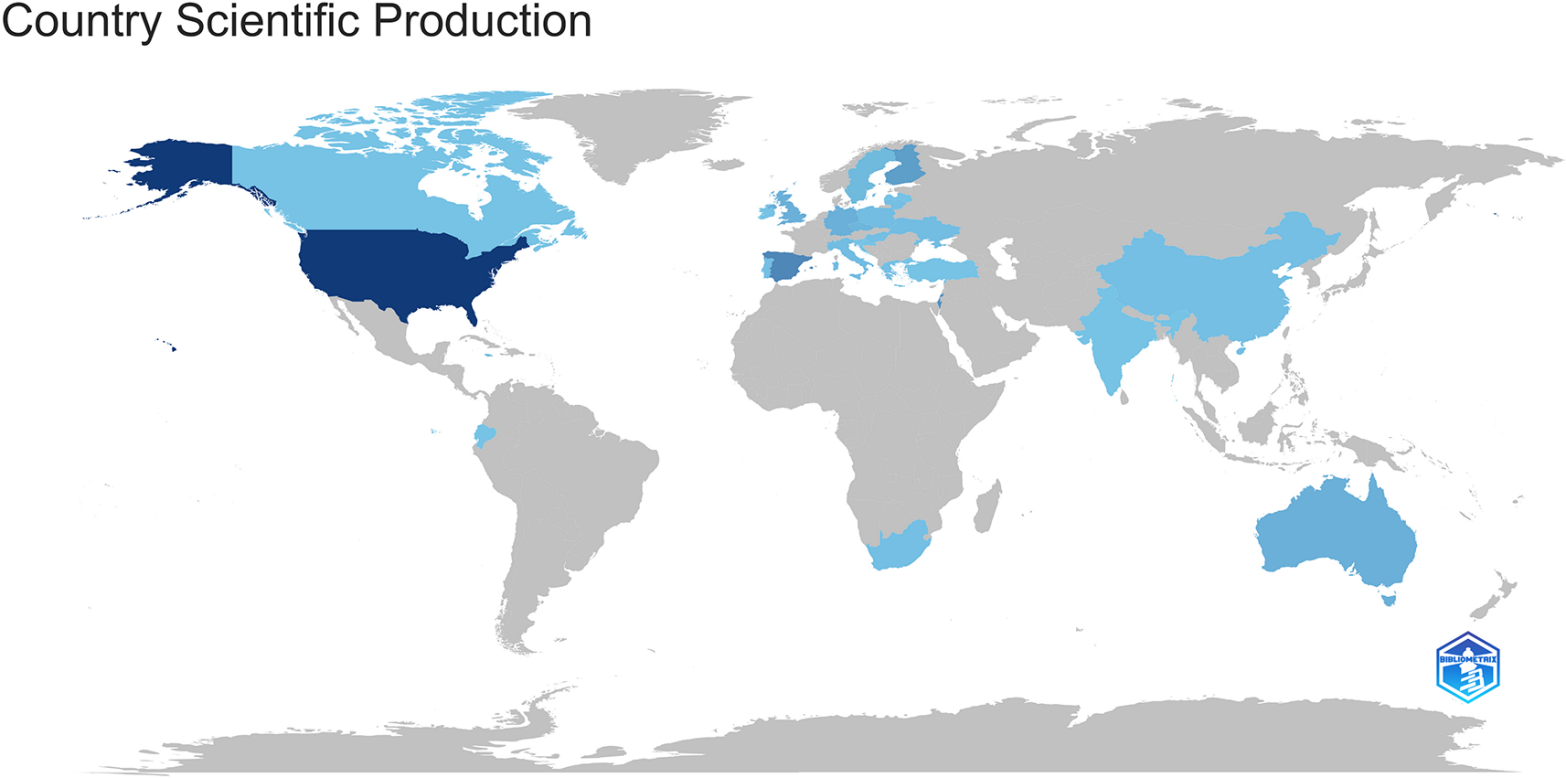
Geographic distribution of scientific output.

This review indicates a pronounced concentration of research in the subject of IE within teacher training across several regions. From 2013 to 2024, the United States (USA) notably dominates with the biggest number of publications (15 articles), but Spain, with 11 articles, also constitutes a majority relative to other nations. This may signify a significant interest and investment in emotional intelligence development initiatives in these nations (refer to Figure 2).

Countries such as Israel and Finland each contributed 4 pieces, while Australia, Germany, Poland, Sweden, and the United Kingdom each contributed 2 articles. Countries with a solitary contribution, each comprising one article, include Canada, China, Croatia, Czech Republic, Greece, Hungary, Italy, Norway, Portugal, Thailand, Ukraine, and Ecuador. While leadership is apparent in specific Northern Hemisphere nations, the geographical variety of the publications indicates an increasing global interest in incorporating emotional intelligence into both initial and continuous teacher training. The minimal academic production in Latin America is notable, underscoring the necessity to promote regional research, particularly in educational settings that demand culturally and socially tailored curricula.

### 3.2 Key nodes in EI teacher training

**Figure 3.**
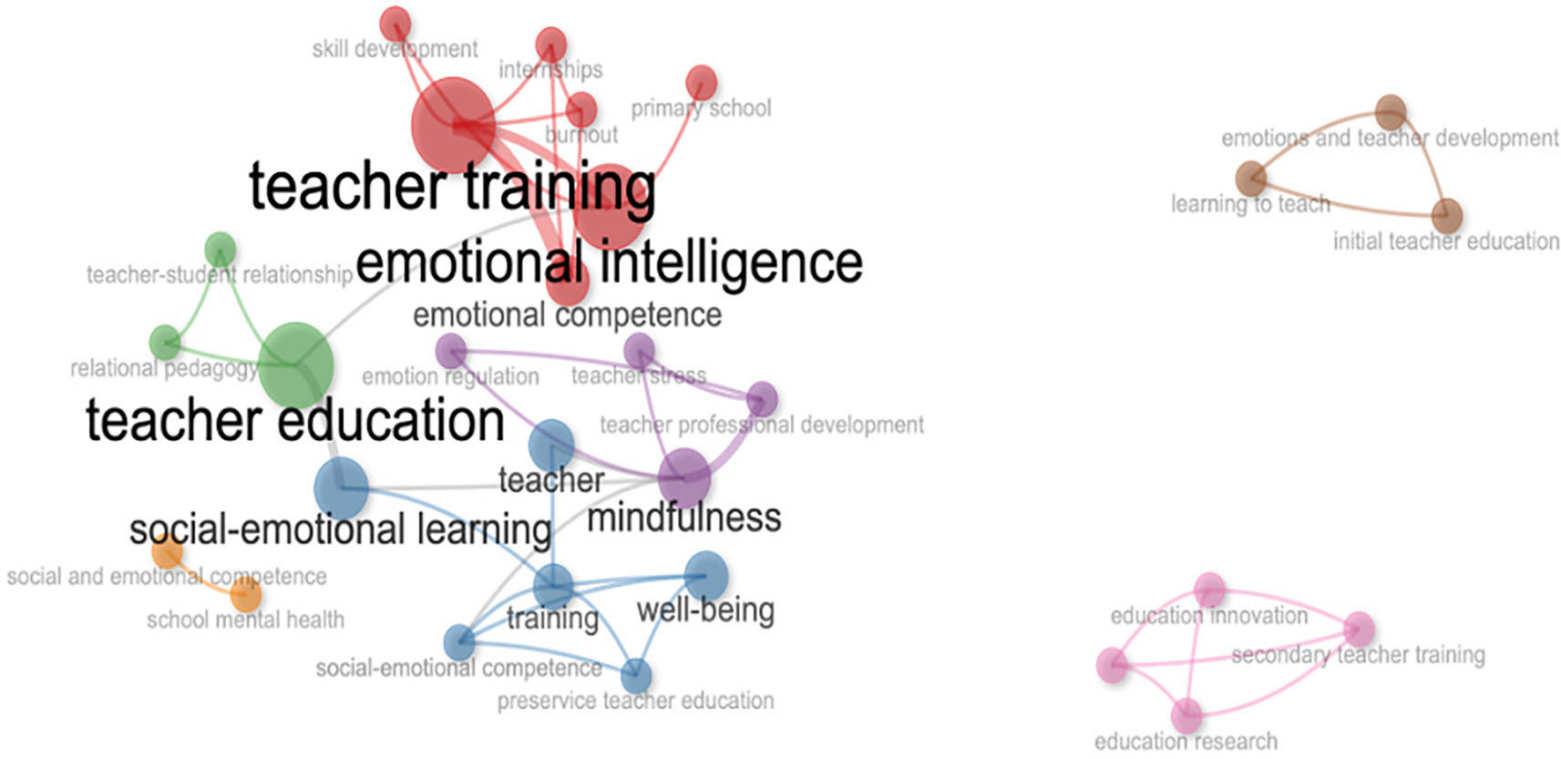
Co-ocurrence authors keywords.

The importance of emotional competences in teacher professional development programs is highlighted by the co-occurrence analysis of studies on emotional intelligence and teacher training, which shows a dense network of related concepts including emotional intelligence, teacher training, and education. Similarly, studies show how emotional management affects teaching, emphasizing its essential role in teachers’ health and stress reduction to support productive professional growth ^40– 43^ (see Figure 3).

The primary ideas include teacher training, emotional intelligence, teacher education, and social-emotional learning, demonstrating a significant alignment between pedagogical instruction and the cultivation of emotional competencies. These concepts are categorized alongside mindfulness, well-being, emotional competence, and emotion regulation, indicating an increasing interest in holistic training methodologies ^41, 44^ that integrate socio-emotional skills, self-regulation, and teacher mental health.

The map also illustrates the distribution of emerging subfields in tiny clusters. As an illustration, the terms “initial teacher education,” “learning to teach,” and “emotions and teacher development” indicate a particular emphasis on initial training. Conversely, another group establishes a connection between education policy, teacher training, and education research, linking the phenomenon to institutional and educational policy frameworks. This thematic diversity is indicative of a shift in the field from individual interventions to more systemic and strategic models, which are designed to enhance the quality of education by addressing the affective underpinnings of teaching. ^40^

### 3.3 Teacher training evolution and dynamics in IE

**Figure 4.**
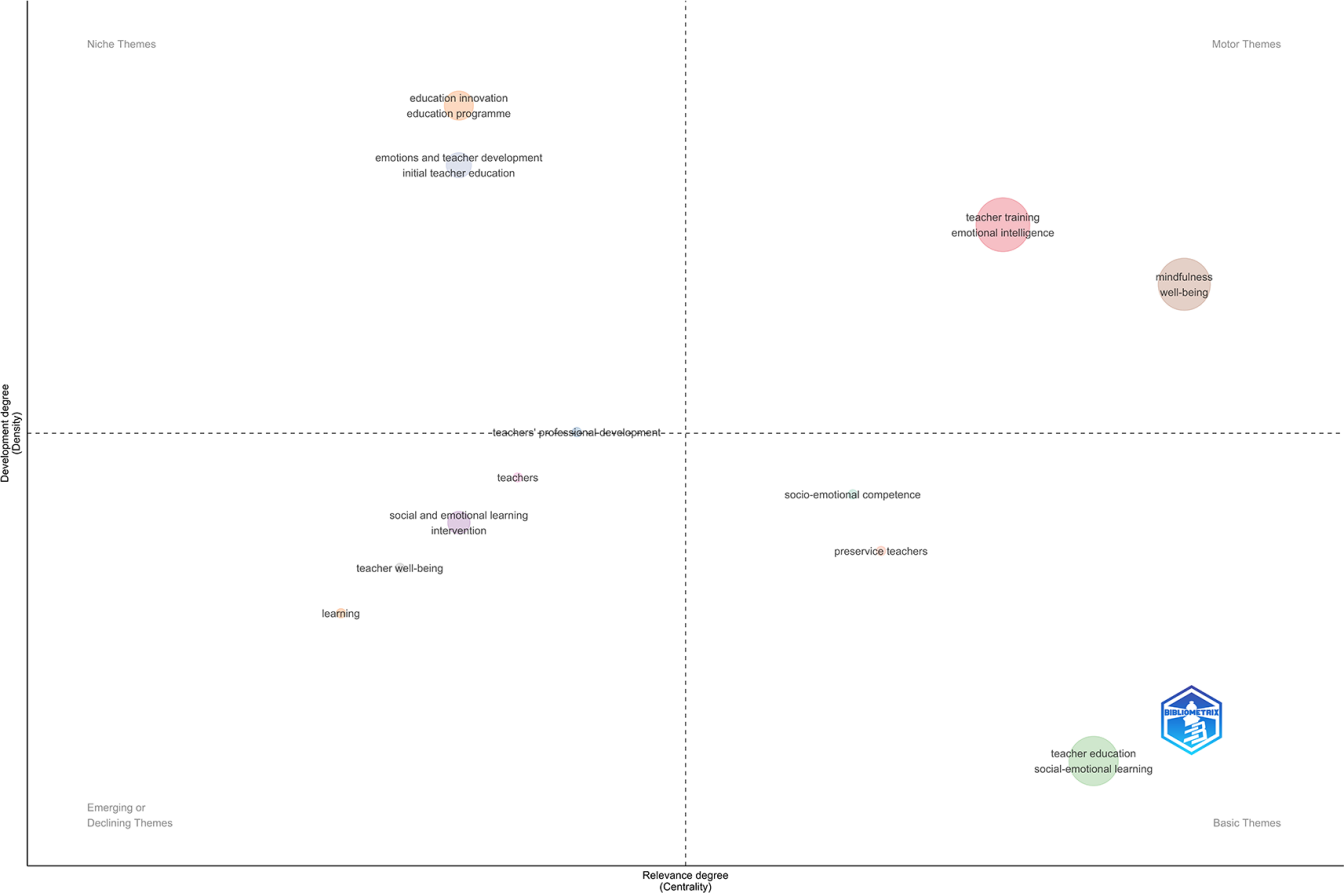
Thematic map.

This section outlines the evolution and current significance of strategic topics in teacher training related to emotional intelligence (see Figure 4). Well-being and mindfulness are crucial components in programs aimed at enhancing teacher self-regulation. ^45^

Topics characterized by low density yet high centrality, including teacher education, social-emotional learning, and professional development, while essential, necessitate enhanced conceptual and methodological refinement to fortify their approaches and guarantee their applicability across diverse educational contexts. ^46^ These themes serve as pivotal axis of the subject, however their internal growth remains rudimentary in relation to their structural significance.

Conversely, niche topics, including education innovation, education programs, and initial teacher education, are identified. These topics exhibit thematic specialization and a certain level of development, but they have minimal connection to central debates in the field, which restricts their systemic impact. ^47^ Despite the fact that these lines are active, they must be connected to the primary axes of emotional intelligence in order to achieve increased visibility and practical application.

On the other hand, subjects including intervention, teacher-student relationships, and teacher professional development display low centrality and density, potentially indicating diminished interest or regions currently undergoing consolidation. Nonetheless, their transformative potential in educational practice indicates that they could be reengaged from novel formative or investigative viewpoints. The field’s diversity and dynamism are affirmed, since it encompasses both established domains and those that are emerging or remain marginal. ^48^

### 3.4 Trends and new directions in IE teacher education

**Figure 5.**
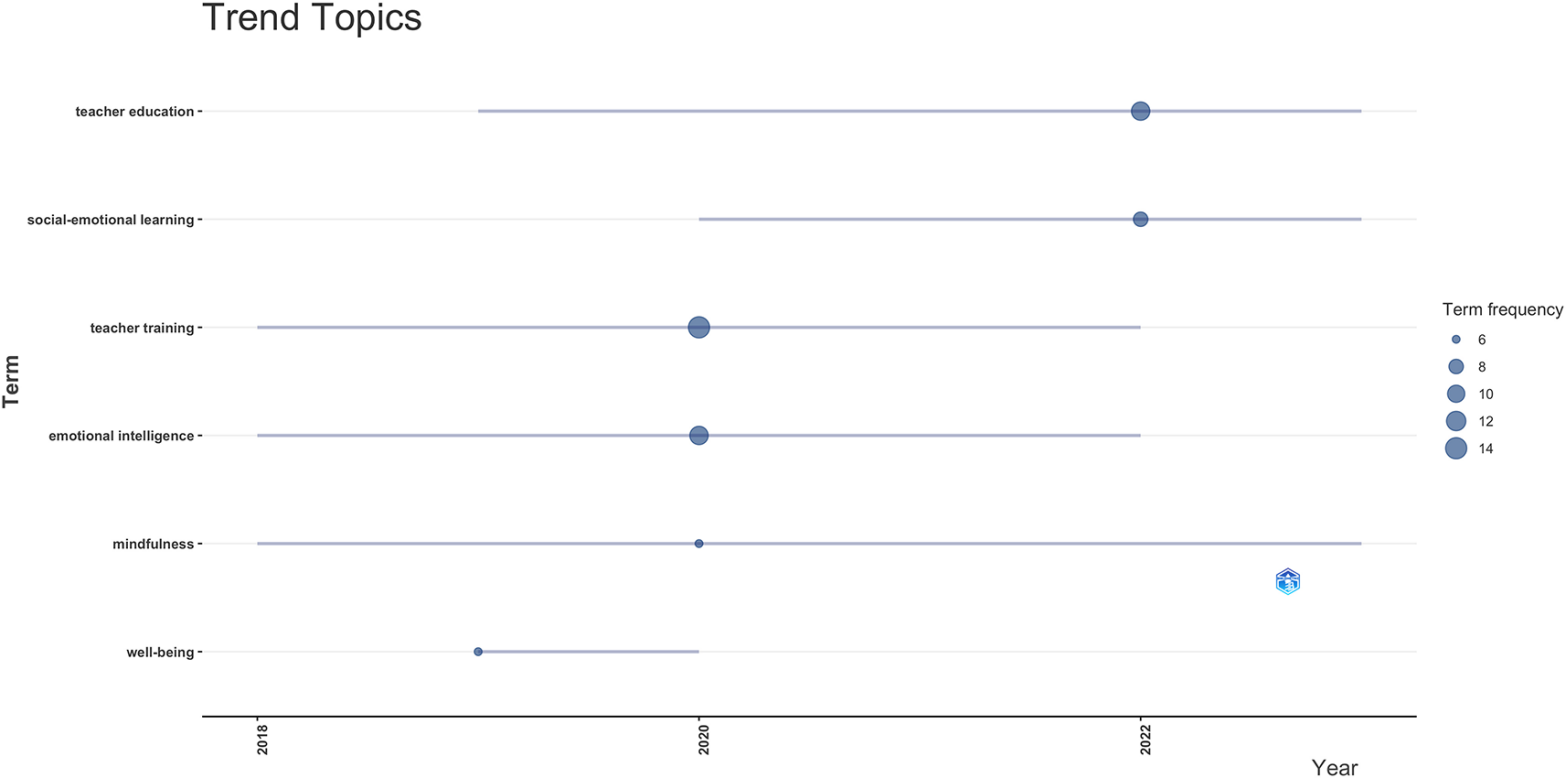
Trend topics.

The examined data exposes important patterns in the body of research on emotional intelligence and teacher preparation (see Figure 5). With 14 and 11 mentions respectively, teacher training and emotional intelligence stand out as main axis in the growth of emotional competences within teacher preparation. Their continuity over time emphasizes their conceptual and pragmatic weight on the present research agenda.

Teacher education and social-emotional learning, with 11 and 8 mentions respectively, demonstrate a consistent presence through 2024, indicating their growing incorporation into training programs and alignment with pedagogical frameworks focused on the holistic development of educators. ^49^ Similarly, mindfulness and well-being, each cited six times, have emerged as topics of contemporary interest associated with the enhancement of emotional well-being in educational settings.

Collectively, these developments signify a persistent evolution in the domain, advancing towards a more holistic perspective of pedagogical practice, whereby emotional development is regarded as a fundamental element in both initial training and professional growth. ^46, 50^

### 3.5 Cooperation and research in the field

**Figure 6.**
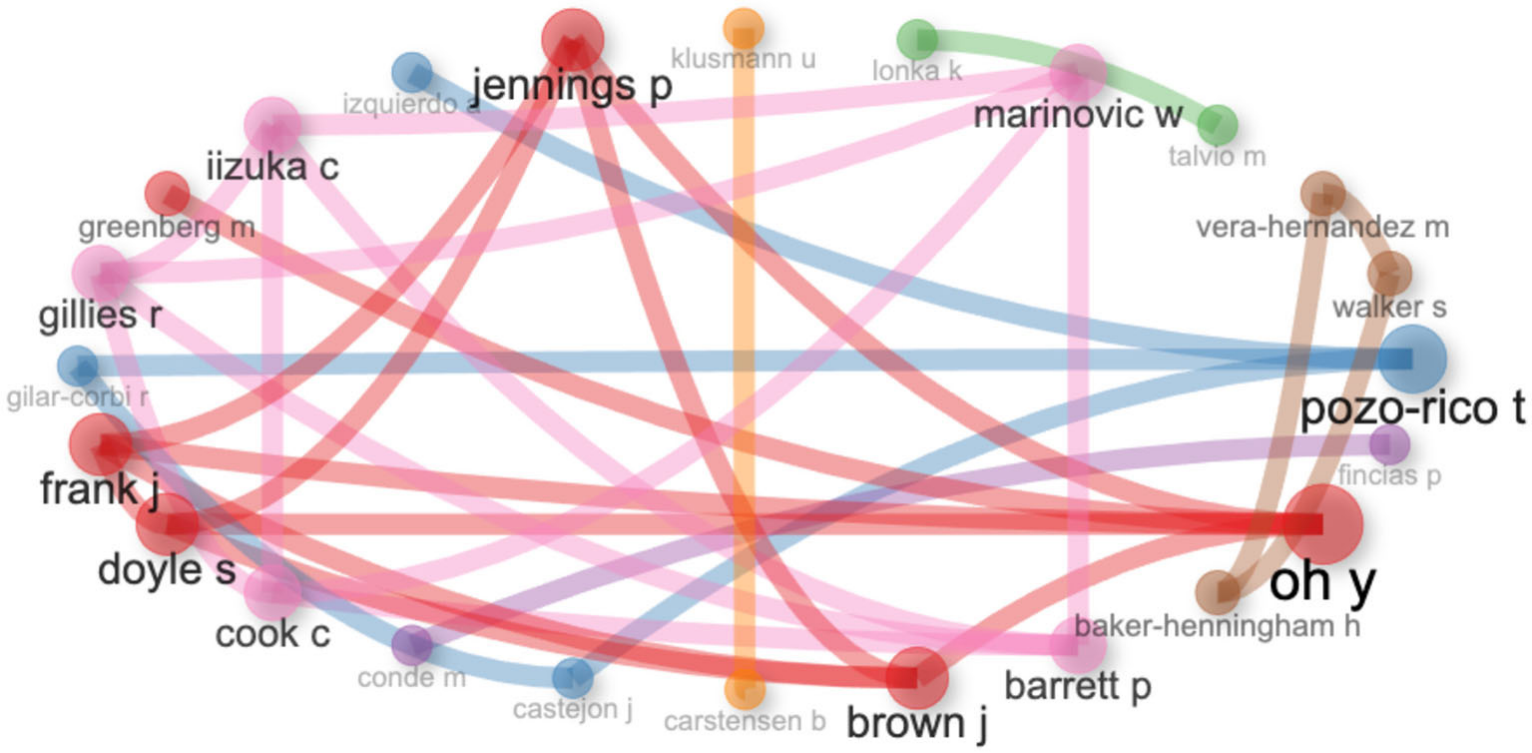
Academic collaboration networks.

The analysis of collaboration networks among authors allows for the identification of key interconnections in the scientific production on emotional intelligence in teacher training (see Figure 6). Consolidated collaboration structures are highlighted, in which certain authors act as central nodes in the mediation and dissemination of knowledge. In particular, Pozo-Rico, Brown, Oh, and Barrett position themselves as prominent figures in the network, suggesting their strategic role in the integration of approaches and the articulation of different research currents in this field.

The closeness among players such as Baker-Henningham, Vera-Hernández, and Alderman signifies a unified network of direct and effective communication, promoting the swift exchange of ideas, outcomes, and conceptual frameworks. This form of dense connectivity is particularly pertinent in interdisciplinary domains such as emotional education, where active collaboration fosters both theoretical and practical progress.

On the other hand, authors such as Carstensen, Klusmann, Fincias, and Conde stand out as important nodes that, despite having fewer relationships, hold a key place as possible opinion leaders. Their presence shows that they can help guide new lines of study and find solutions to problems that are happening now in the area of teacher emotional training. This collaboration network shows how the area is slowly coming together, showing both the shared work and the different study teams from different places and institutions.

### 3.6 Results addressing question 1: What strategies and programs for teacher training in emotional intelligence have been implemented from 2013 to 2024?

Over the past decade, teacher training in emotional intelligence has become crucial, emphasizing the development of social-emotional competencies that impact teacher well-being and foster healthier classroom environments. The implemented strategies and programs encompass various approaches, including mindfulness-based interventions and innovative educational methodologies. ^51^ The following outlines key strategies and programs in this field.

***3.6.1 Mindfulness and SEL: A powerful combination in teacher emotional training***

Mindfulness programs combined with Social and Emotional Learning (SEL) improve emotional teacher training, especially in self-awareness, self-regulation, and emotional management. Jones and Doolittle ^52^ say SEL promotes emotional competences like empathy, responsible decision-making, and good relationships. Mindfulness in these programs improves SEL, ^53^ helping instructors focus, regulate emotions, and handle classroom issues.

Mindfulness improves emotional self-regulation, helping teachers handle stress in a changing educational environment. Research shows that Mindfulness-Based Programs (MBP) and Tuning in to Kids improve teachers’ emotional skills and resilience in stressful situations. ^54^ According to Meiklejohn et al., ^45^ mindfulness training for teachers increases their emotional well-being and student interaction management, creating a happy learning environment. This combination improves teachers’ emotional control and lesson quality and environment.

***3.6.2 Integration of online models for SEL training***

The digitization of education has transformed access to EI training for teachers, providing new opportunities via virtual environments. The integration of SEL in these models has shown benefits, enabling teachers to develop essential skills like self-management and empathy interactively and flexibly. ^55^ Virtual environments enable teachers to engage in simulations that mimic real educational scenarios, offering a safe space for reflection on emotional responses and enhancement of emotional regulation skills. ^56, 57^

Online learning platforms facilitate ongoing reflection and feedback, essential for teachers to assess their development in social-emotional competencies. ^58^ These tools facilitate repeated practice and self-assessment, essential for incorporating SEL skills into teaching. Virtual simulations are an effective strategy for preparing teachers for complex situations, offering practical experience in applying social-emotional strategies in their work environment. This enhances emotional well-being and boosts confidence in managing classroom dynamics effectively.

***3.6.3 Emotion diaries: An essential resource for emotional intelligence***

Teacher training programmes that promote self-awareness and emotional understanding use emotion journals. ^24^ Daily reflective writing helps teachers recognise and evaluate their emotions in diverse settings, improving their understanding of how they affect classroom performance and student interactions. ^59^ Self-reflection helps teachers regulate emotions in high-stress situations by identifying emotional patterns. ^60^

Emotion journals enhance empathy and understanding. Teacher reflection on emotional experiences helps them comprehend classroom dynamics. This knowledge helps teachers manage relationships and assist children’ emotional growth in a collaborative and empathic classroom. Bravo-Sánchez ^46^ notes that documenting and reflecting on feelings helps teachers create an emotionally healthy learning environment, enhancing school morale and productivity.

Emotional diaries help teachers self-assess their emotional development. ^24^ Reviewing their entries helps individuals discover emotional regulation improvements and issues. ^61^ This lays the groundwork for individualized measures to improve emotional coping and classroom well-being. ^24^

***3.6.4 Brief programs***

Short-term teacher training programs, lasting 2 to 7 weeks, effectively enhance emotional competencies, though their influence on student academic performance is still debated. ^26^ These programs enable teachers to quickly gain essential tools for managing emotions and promoting a healthy learning environment. ^62^ Concerns exist regarding the sustainability of these interventions and their long-term effects, necessitating further research to assess whether enhancements in teacher well-being lead to lasting academic benefits for students. ^63, 64^

***3.6.5 Emotional literacy: Initiatives that enhance educators and learning environments***

Programs aimed at enhancing emotional intelligence in teachers, particularly those with extended duration and ongoing support, have demonstrated beneficial outcomes for personal well-being and the emotional atmosphere in classrooms. Initiatives like EL4VET, CASEL, and CARE have effectively fostered essential emotional competencies via emotional literacy. These programs enhance self-awareness and emotional management, directly influencing the quality of teacher-student interactions. ^14^

### 3.7 Results addressing question 2: What defines an emotional intelligence program for teacher training?

Programs focused on emotional intelligence in teacher training are essential for personal and professional growth. A systematic review of the selected articles revealed common characteristics that enhance the effectiveness of these programs and their impact on teachers’ socio-emotional skills.

***3.7.1 Theoretical and practical components of IE programs***

Effective emotional intelligence programs in teacher training combine theoretical and practical elements to cultivate socio-emotional skills comprehensively and sustainably. ^65^ Essential elements include improving emotional awareness, regulation, empathy, and the ability to establish positive interpersonal relationships. ^66^ Programs generally begin by establishing a solid theoretical foundation, focusing on understanding personal and others’ emotions and analyzing their emotional impact on classroom dynamics.

Mindfulness and conscious reflection are recommended to improve self-regulation, self-care, and emotional resilience in teachers. Mindfulness is a crucial approach for managing stress and improving awareness, directly influencing teaching quality and the emotional climate of the classroom. Assertive communication and conflict resolution techniques are examined, enabling effective management of complex situations. ^67^

The programs enhance teachers’ emotional understanding and management, enabling them to recognize and respond to students’ emotions, thus fostering empathetic interactions and promoting an inclusive learning environment. ^24^ Ongoing monitoring, feedback, and practice are essential for maintaining and applying emotional competencies acquired during training in professional settings.

***3.7.2 Incorporation of novel educational methods for EI***

Innovative educational methodologies in teacher training programs improve teachers’ emotional well-being and skills. ^68^ These methodologies go beyond traditional instruction, incorporating strategies that promote active, participatory, and reflective learning about emotions and interpersonal relationships in the classroom. ^69^ Project-Based Learning (PBL) allows teachers to collaboratively tackle real or simulated problems, enhancing skills such as empathy, collaboration, and emotional self-regulation. ^70^

Simulation-based learning is essential for developing emotional competencies in realistic, controlled settings. Educators participate in both in-person and virtual simulations that address emotionally charged scenarios, such as student conflicts and disciplinary matters. ^57^ This experience improves decision-making, stress management, and emotional regulation. ^56^ Online simulations offer considerable flexibility, allowing teachers to practice and receive feedback in a secure, controlled environment. ^71^

Reflection-Based Learning exemplifies an innovative approach, wherein educators record their emotions in diaries to assess their influence on classroom dynamics and student interactions. This methodology improves emotional self-awareness and helps teachers identify emotional patterns affecting their educational practice. ^72^ The integration of these methods with community engagement facilitates experience and strategy exchange, creating a supportive environment for teachers to collaboratively enhance their emotional competencies. ^41^

***3.7.3 Self-awareness and self-regulation training***

Self-awareness and emotional self-regulation training are essential components of teacher education programs, as these skills are vital for handling the profession’s emotional demands. ^26^ These interventions, aimed at recognizing and managing emotions, positively influence teacher resilience and effectiveness. Self-awareness enables teachers to identify their emotional responses in challenging situations, facilitating better behavior regulation and adaptive responses. ^73^

Emotional self-regulation during stress is essential for teachers’ well-being and fostering a positive learning environment. ^74^ Training programs equip teachers with essential tools to handle the stress and frustrations of teaching, leading to improved student interactions and enhanced classroom stability. ^75^ Studies indicate that enhancing these skills improves teachers’ emotional well-being and indirectly benefits students’ academic performance by fostering a more positive school climate. ^53^

***3.7.4 Adaptation to online learning settings***

The adaptation of emotional intelligence training programs to virtual platforms is a crucial aspect in today’s digitalized educational landscape. ^29, 55^ Virtual teaching not only facilitates access to training but also offers new opportunities for teachers to develop emotional competencies in an interactive and contextualized way. ^76^ Programs utilizing digital tools, including online simulations and collaborative platforms, are as effective as in-person methods in developing socio-emotional skills like self-regulation, empathy, and stress management. ^24, 77^

Virtual environments offer personalized and flexible experiences tailored to the specific needs of educators. Online simulations allow participants to engage with and analyze complex scenarios. ^57^ Simulations, along with self-reflection exercises and group feedback, are comparable to face-to-face sessions in fostering emotional competencies, while allowing for the repetition and revision of exercises as necessary. ^57, 76^

Online collaborative learning platforms enable real-time interaction among teachers, fostering experience exchange and the development of practice communities that support sustained emotional and professional growth. ^16, 78^ Virtual communities offer emotional and professional support, enhancing the incorporation of emotional skills in education. Adapting programs to virtual environments enhances accessibility and relevance in educational technology. ^79, 80^

***3.7.5 Programs with a flexible duration and structure***

Diverse in duration and structure, emotional intelligence training programs can be easily adapted to various educational contexts. The flexible duration enables teachers to engage with these programs based on their needs and availability, facilitating emotional skill development even in time-limited situations. ^12^

***3.7.6 Impact on well-being and emotional efficacy***

Emotional intelligence programs for teacher training significantly enhance teachers’ overall well-being. ^54^ These programs aim to enhance emotional competencies, promote mental health balance, and strengthen resilience to daily school demands. ^81^ Teaching strategies like stress management, emotional self-regulation, and enhancing emotional awareness equip educators to handle the demands of their profession, reducing emotional exhaustion and burnout symptoms. ^26^

These programs enhance self-efficacy and professional confidence, leading to a greater sense of control over the work environment and improved management of challenging situations. ^82^ The positive effect on teacher well-being enhances job satisfaction and quality of life, contributing to the development of collaborative learning environments. ^83, 84^

***3.7.7 Continuous support and sustainability***

Ongoing support mechanisms are crucial in emotional intelligence training for teachers. Initial training alone is inadequate for developing emotional competencies; a support system is essential for teachers to effectively consolidate and apply these skills in real educational contexts. ^78, 82^ Studies show that programs with continuous follow-up components, such as regular feedback, mentoring, and community access, significantly improve the retention and enhancement of skills. ^28, 51, 81^

Ongoing mentoring fosters reflection and adaptation of emotional strategies to meet the distinct challenges teachers face in their daily practice. ^85^ Communities of practice provide a venue for experience sharing and peer support, facilitating a safe space for exploring self-regulation and stress management strategies. ^59^ These communities allow participants to evaluate and adjust their emotional strategies, promoting collaborative learning that improves program sustainability. ^32^

Personalized mentoring is essential for developing emotional competencies, offering expert guidance to tackle individual challenges, refine goals, and evaluate progress consistently. Supportive elements enhance emotional competencies, integral to teachers’ professional identity, thereby improving their well-being and that of the educational community over time. ^63^ Sustainability strategies frame emotional intelligence programs as vital elements of teachers’ professional development, rather than as standalone interventions.

***3.7.8 Measurement is essential***

This review outlines measurement tools, such as questionnaires, scales, and tests, for assessing emotional intelligence in teacher training.

Among the tools used were: a) EQ-i (Emotional Quotient Inventory), which consists of a self-reported questionnaire that measures emotional competencies in several dimensions; b) The MSCEIT test (Mayer-Salovey-Caruso Emotional Intelligence Test), which is an assessment tool based on the ability to measure emotional intelligence as a set of skills; c) the TESC scale (Teacher Emotional Self-Competency Scale), used to measure the emotional competence of teachers; d) CPI (Classroom Performance Inventory) which relates the emotional competence of teachers with classroom performance; e) The SEL Competency Checklist, which is a tool to measure competencies in social-emotional learning in teachers, specifically in educational contexts; f ) The TMMS (Trait Meta-Mood Scale), a self-assessment scale that measures emotional clarity, emotional attention and emotional repair; g) The WLEIS (Wong and Law Emotional Intelligence Scale), used to measure emotional intelligence through four dimensions: self-regulation, emotional perception, use of emotions and emotional facilitation; h) Emotional Competence Inventory (ECI), a tool based on self-assessment and peer assessment to measure emotional competence in an organizational environment.

The systematic review showed that most studies using these tools report reliability indices (Cronbach’s alpha) generally above 0.80, indicating strong internal consistency. Contextual validation is sometimes deemed essential depending on the teaching population.

The research indicates improved student performance and enhanced emotional well-being among teachers. The cultivation of emotional competencies in teachers enhances their professional lives and improves student performance, creating a positive cycle in education.

## 4. Discussion

This study investigated teacher emotional intelligence training programs from 2013 to 2024. Training programs emphasize teachers’ personal and professional well-being, the positive association between instructors’ socio-emotional competencies and students’ academic success, and a person-centered approach, according to a comprehensive literature analysis. Valente & Lourenço, ^86^ Fernández Berrocal et al., ^87^ and Nóbrega et al. ^88^ support this. These studies show that emotional intelligence improves classroom dynamics and promotes collaborative, empathic learning. ^1, 28^ It improves instructors’ and students’ emotional, social, and mental health by helping them understand their surroundings and make educated decisions in daily disputes. ^89^

Between 2013 and 2024, teacher training strategies in emotional intelligence significantly enhanced academic output in the educational sector. This growth, especially in recent years, reflects the field’s maturation and a rising interest in its practical application. Research in the US, Spain, and Europe remains prominent, yet recent years have witnessed growing involvement from emerging regions such as Asia and Latin America, underscoring a global interest in improving emotional competencies in education. ^29, 84, 87^

Key concepts include self-awareness and self-regulation, essential for emotional management in the classroom, as well as the development of empathy and interpersonal skills. Theoretical models, such as the social-cognitive approach and Mayer and Salovey’s ability model, highlight the interdependence of individual emotions and their sociocultural context. ^23, 25^ Research shows that emotional intelligence affects teacher well-being, academic performance, and the emotional climate of the classroom, as teachers with emotional skills create a positive and safe learning environment for students.

Research on emotional intelligence has provided theoretical-methodological methodologies, models, and verified testing procedures. ^90^ Trends in education emphasize innovative methods and medium-term programs that educate socio-emotional skills. Effective stress management involves emotional control, self-regulation, empathy, responsible decision-making, positive connections, and resilience. ^52– 54^ These programs improved teachers’ emotional well-being and ability to manage student relationships, which affects teaching quality and classroom climate.

Online simulations and digital technology increase introspection and emotional self-regulation in realistic classrooms. This strategy helps modern classrooms by offering a safe space for reflection, emotional feedback, and student and teacher emotional management. These methods offer exciting research pathways.

Studies on emotional intelligence programs for teacher training and promoting a positive classroom climate ^53, 66, 67, 69, 75^ highlight the following characteristics: a) Strong theoretical foundations on the various categories, programs, and methodologies of emotional intelligence; b) Purposes: development of teachers’ emotional competence for understanding and managing their own and their students’ emotions; c) Integration of categories like strengthening emotional awareness and self-awareness, empathy, self-regulation, self-care, emotional resilience, stress management, mindfulness, and the ability ICTs and associated resources, platforms, and virtual environments increase accessibility and reach; d) Results: a positive, emotionally healthy, and balanced school climate, stress and frustration management, better student interaction, improved teacher and student well-being, and academic performance; e ) Durability: diverse, flexible, and adaptable to different contexts; f) Sustainability: educational and institutional policies on emotional intelligence, innovative programs that interrelate and integrate.

The review suggests that social and emotional learning (SEL) must systematically integrate cognition, emotion, and behavior in daily life to help students regulate emotions, make responsible decisions, and form positive relationships. EI training should balance kids’ immediate benefits and teachers’ well-being. Educators need this dual approach to improve emotional competences, classroom performance, mental health, and job happiness. Future prospects suggest expanding EI training to virtual platforms to promote access and sustainability in varied educational environments. ^46, 77^

## 5. Conclusions

The current study systematically analyzed emotional intelligence training programs for teachers from 2013 to 2024, highlighting a comprehensive approach that prioritizes educators’ personal and professional well-being and establishes a positive correlation between teachers’ socio-emotional competencies and students’ academic performance using person-centered methodologies. A thorough literature review supports this viewpoint with the contributions of Valente & Lourenço, ^86^ Fernández Berrocal, ^87^ and Nóbrega, ^88^ who show that developing emotional intelligence significantly improves classroom dynamics and fosters collaborative and empathetic learning environments. ^1, 28^ These findings indicate that such competencies improve the emotional, social, and mental health of both teachers and students, allowing for a better understanding of the educational context and promoting informed decision-making in everyday conflicts. ^89^

The 2013-2024 period analysis demonstrates a significant enhancement in academic performance in the education sector, which is attributed to the implementation of teacher training strategies in emotional intelligence. This improvement is indicative of the maturation of the discipline and the increasing interest in its pragmatic application. Although the United States, Spain, and Europe continue to dominate research, there is a growing interest in the development of emotional competencies in educational settings, particularly in regions such as Asia and Latin America. ^29, 84, 87^ Self-awareness and self-regulation are essential components of pedagogical emotional management, as well as the development of interpersonal skills and empathy. These are the fundamental constructs. The socio-cognitive approach and the Mayer and Salovey skills model, which are the most prevalent theoretical frameworks, emphasize the interdependence between individual emotions and their sociocultural context. ^23, 25^ This relationship establishes that teacher emotional intelligence is a determining factor for faculty well-being, academic performance, and classroom emotional climate, thereby creating safe and positive environments in both the workplace and the learning environment.

## 6. Limitations

This section is not mandatory but may be added if there are patents resulting from the work reported in this manuscript. This systematic review has inherent limitations that may affect the results. This bibliometric methodology offers valuable insights into trends and developments in the literature regarding emotional intelligence training for teachers. This methodology prioritizes citation frequency and patterns, possibly neglecting less cited but pertinent studies. This review may not cover all important contributions to knowledge, especially those related to alternative or emerging approaches.

The review analyzed two major databases, Scopus and Web of Science, recognized for their quality and thematic scope. The databases include substantial peer-reviewed research; however, their coverage is limited, and important studies, especially from regional sources or grey literature, may be excluded. This review focuses solely on articles from academic journals, excluding other formats such as book chapters, conference papers, and theses that could offer important insights into teachers’ emotional development.

An author co-citation analysis was performed to overcome these limitations, facilitating the expansion of the knowledge base by identifying referenced works in the selected articles. This method allows for the inclusion of significant works in the field, irrespective of their direct presence in the reviewed articles. The review included 63 empirical studies selected through a rigorous process to ensure relevance to the study objectives, enhancing the reliability and validity of the results.

This review provides a thorough overview of significant trends and features in emotional intelligence training programs for teachers, despite some methodological and scope limitations. The study offers a pertinent overview of the field’s current status and future directions, despite its limitations.

## Data Availability

M. Aponte-Moreno, A. D. Vargas Sánchez, & M. Arnao Vásquez. Data Extender - A systematic review [Data set]. Zenodo. 2025. Doi: https://doi.org/10.5281/zenodo.15832862 ^38^ The project contains the following extended data:
1.

**Data Article review 2024_1 english.xlsx**

2.

**
PRISMA_2020_checklist_Art.1.docx**

3.

**
Figure 1_PRISMA_flow_diagram_Art_2024.jpg**

4.

**
Figure 2_Geographic Distribution of Scientific Output.jpg**

5.

**
Figure 3_Co-ocurrence_authors_keywords.jpg**

6.

**
Figure 4_ThematicMap.jpg**

7.

**
Figure 5_TrendTopics.jpg**

8.

**
Figure 6_Academic_Collaboration_Network.jpg** Creative Commons Attribution 4.0 International
