# Supplementary material for: The effects of emotional intelligence training programs on educators: a systematic review
Source: F1000Res. 2025 Aug 29;14:842. [Version 1] doi: 10.12688/f1000research.166190.1 (PMC12457898; doi:10.12688/f1000research.166190.1)
Supplement: Peer review history

# Reviewer report: Reviewer response for version 1

**DOI:** 10.5256/f1000research.183146.r410809

**Published:** 2025-09-23

**Authors:** Nelly Gromiria Lagos San Martín

**Affiliations:** 1 University of Bío-Bío, Chillán, Chile

I find this work interesting. It is very well structured and follows a logical order. It is organized using the PRISMA method and perfectly meets the requirements of a rigorous systematic review. It sets an appropriate objective for the research, thoroughly analyzes a considerable number of texts, and presents the analyses excellently, both graphically and textually, which facilitates understanding. The discussion and conclusions are consistent with the objective of the work. Finally, it is a comprehensive study based on a large number of relevant and up-to-date references, which give the study great argumentative weight.

Are the rationale for, and objectives of, the Systematic Review clearly stated?

Yes

Is the statistical analysis and its interpretation appropriate?

Yes

If this is a Living Systematic Review, is the ‘living’ method appropriate and is the search schedule clearly defined and justified? (‘Living Systematic Review’ or a variation of this term should be included in the title.)

Yes

Are sufficient details of the methods and analysis provided to allow replication by others?

Yes

Are the conclusions drawn adequately supported by the results presented in the review?

Yes

Reviewer Expertise:

I think the work is interesting as it is.

I confirm that I have read this submission and believe that I have an appropriate level of expertise to confirm that it is of an acceptable scientific standard.

# Reviewer report: Reviewer response for version 1

**DOI:** 10.5256/f1000research.183146.r410813

**Published:** 2025-09-05

**Authors:** Juanita Pedraza

**Affiliations:** 1 University Carlos III de Madrid, Madrid, Spain

This article under review addresses a topic of great relevance: the development of emotional competencies in teachers. A literature review was conducted for the period between 2013 and 2024, using the Scopus and Web of Science databases as references. The research is based on answering two specific questions: What strategies and training programs for emotional competencies have been implemented during the study period? and What are the characteristics of such programs or strategies?

 The article highlights the usefulness of techniques such as simulations and the emotional diary, while also revealing the effectiveness of progressive training using technological resources.

 Regarding evaluation, several methods are cited that, among other things, allow for the verification that training in these types of competencies not only improves the work environment but also positively impacts the classroom. These competencies benefit the emotional well-being of teachers and are transmitted to students, strengthening the virtuous cycle of training in these competencies.

 Based on this review, a promising future line of research would be to specify the factors that ensure process feedback, thereby enhancing the integration of these strategies into teacher training programs..

Are the rationale for, and objectives of, the Systematic Review clearly stated?

Yes

Is the statistical analysis and its interpretation appropriate?

Yes

If this is a Living Systematic Review, is the ‘living’ method appropriate and is the search schedule clearly defined and justified? (‘Living Systematic Review’ or a variation of this term should be included in the title.)

Not applicable

Are sufficient details of the methods and analysis provided to allow replication by others?

Yes

Are the conclusions drawn adequately supported by the results presented in the review?

Yes

Reviewer Expertise:

Education for global citizenship

I confirm that I have read this submission and believe that I have an appropriate level of expertise to confirm that it is of an acceptable scientific standard.
